# Catalytic Mechanism Investigation of Lysine-Specific Demethylase 1 (LSD1): A Computational Study

**DOI:** 10.1371/journal.pone.0025444

**Published:** 2011-09-30

**Authors:** Xiangqian Kong, Sisheng Ouyang, Zhongjie Liang, Junyan Lu, Liang Chen, Bairong Shen, Donghai Li, Mingyue Zheng, Keqin Kathy Li, Cheng Luo, Hualiang Jiang

**Affiliations:** 1 State Key Laboratory of Drug Research, Drug Discovery and Design Center, Shanghai Institute of Materia Medica, Chinese Academy of Sciences, Shanghai, China; 2 Center for Systems Biology, Soochow University, Jiangsu, China; 3 State Key Laboratory of Pharmaceutical Biotechnology, School of Life Sciences, Jiangsu Diabetes Research Center, Nanjing University, Nanjing, China; 4 State Key Laboratory of Medical Genomics, Shanghai Institute of Hematology, Rui Jin Hospital, Shanghai Jiao Tong University School of Medicine, Shanghai, China; 5 School of Pharmacy, East China University of Science and Technology, Shanghai, China; National Institute for Medical Research, Medical Research Council, United Kingdom

## Abstract

Lysine-specific demethylase 1 (LSD1), the first identified histone demethylase, is a flavin-dependent amine oxidase which specifically demethylates mono- or dimethylated H3K4 and H3K9 via a redox process. It participates in a broad spectrum of biological processes and is of high importance in cell proliferation, adipogenesis, spermatogenesis, chromosome segregation and embryonic development. To date, as a potential drug target for discovering anti-tumor drugs, the medical significance of LSD1 has been greatly appreciated. However, the catalytic mechanism for the rate-limiting reductive half-reaction in demethylation remains controversial. By employing a combined computational approach including molecular modeling, molecular dynamics (MD) simulations and quantum mechanics/molecular mechanics (QM/MM) calculations, the catalytic mechanism of dimethylated H3K4 demethylation by LSD1 was characterized in details. The three-dimensional (3D) model of the complex was composed of LSD1, CoREST, and histone substrate. A 30-ns MD simulation of the model highlights the pivotal role of the conserved Tyr761 and lysine-water-flavin motif in properly orienting flavin adenine dinucleotide (FAD) with respect to substrate. The synergy of the two factors effectively stabilizes the catalytic environment and facilitated the demethylation reaction. On the basis of the reasonable consistence between simulation results and available mutagenesis data, QM/MM strategy was further employed to probe the catalytic mechanism of the reductive half-reaction in demethylation. The characteristics of the demethylation pathway determined by the potential energy surface and charge distribution analysis indicates that this reaction belongs to the direct hydride transfer mechanism. Our study provides insights into the LSD1 mechanism of reductive half-reaction in demethylation and has important implications for the discovery of regulators against LSD1 enzymes.

## Introduction

Histones are subjected to a variety of post-translational modifications, including acetylation, phosphorylation, ubiquitination, sumolation and methylation [1], [2]. These modifications steadily modulate chromatin structure and the consequent diverse chromatin-based processes in a combined fashion. Distinguished from other modifications that are dynamically regulated, histone methylation has long been thought to be a permanent epigenetic marker. However, the recent discovery of lysine-specific demethylase 1 (LSD1) and Jumonji domain-containing proteins strongly challenged this dogma, by demonstrating that histone lysine methylation can be actively and dynamically regulated by histone methylase and demethylase [3].

**Figure 1:**
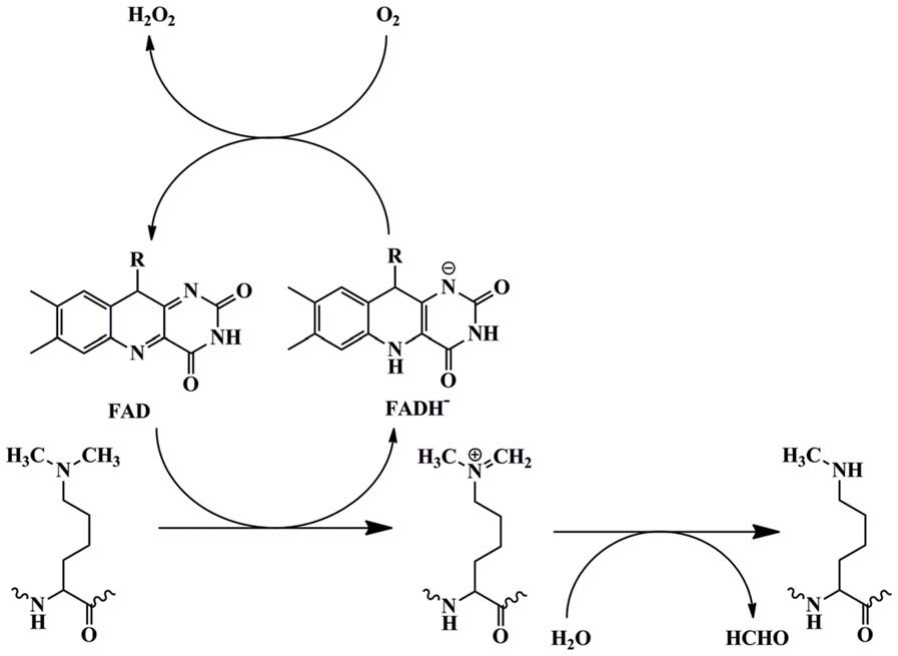
The proposed catalytic mechanism for the overall demethylation reaction of LSD1.

LSD1, the first identified histone demethylase, is a flavin-dependent amine oxidase and conserved from Schizosaccharomyces pombe to mammals [4]. As a member of monoamine oxidase (MAO) family, LSD1 catalyzes the specific demethylation of mono- or dimethylated histone H3 lysine4 (H3K4) and H3 lysine 9 (H3K9) via a redox process. As illustrated in Figure 1, during the reductive half-reaction, the α-CH bond of the substrate was oxidatively cleaved to form an imine intermediate with the coinstantaneous transfer of a hydride equivalent to flavin-adenine dinucleotide (FAD). The reduced cofactor was then reoxidated to its functional form by molecular oxygen accompanied with the release of hydrogen peroxide byproduct during the oxidative half-reaction. The imine intermediate was further hydrolyzed non-enzymatically to release the unmodified lysine and formaldehyde. In addition to histone substrates, LSD1 can also act on non-histone substrates, such as p53 [5], [6], DNMT1 [7], and MYPT1 [8]. Meanwhile, LSD1 was an integral component of miscellaneous chromatin remodeling and transcriptional complexes [9], [10], [11], [12]. The ability to modulate such a wide range of substrates and pathways has implied the pivotal role of LSD1 in a broad spectrum of biological processes, including cell proliferation [13], adipogenesis [14], spermatogenesis [15], chromosome segregation [16], pluripotency regulation of stem cell [17] and embryonic development [18]. The dysregulation of LSD1 activity possesses a significant impact on human carcinogenesis [8] and has been implicated in maintenance of a variety of cancer types, such as neuroblastoma [19], breast cancer [20], [21], colon cancer [22], etc. Moreover, the expression of LSD1 is closely correlated with the relapse of prostate cancer during therapy [23], [24].

**Figure 2:**
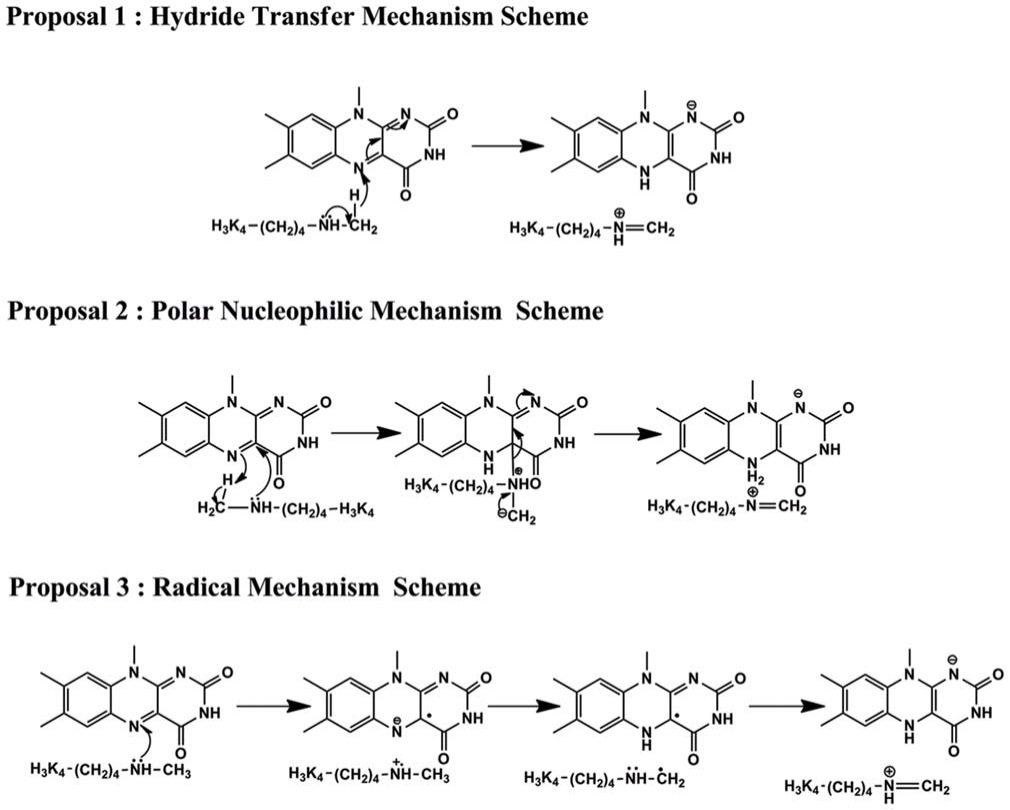
Three major suggested catalytic mechanism proposals for the reductive half-reaction of the flavin-dependent amine oxidases.

However, for most of the flavin-dependent amine oxidases, a major unraveled portion of the chemical mechanism is the reductive half-reaction which involves the irreversible CH bond cleavage. There are three major protracted controversies for this issue which include hydride transfer mechanism [25], polar nucleophilic mechanism [26] and radical mechanism [27]. As illustrated in Figure 2, hydride transfer mechanism may be the most unequivocal process which only involves a direct transfer of a hydride from the substrate α-carbon to flavin, while the polar nucleophilic mechanism and radical mechanism necessitate an adduct intermediate and radical intermediate preceding the CH cleavage, respectively. Due to the significantly conserved architecture of the catalytic domain and the chemical nature of catalytic reaction, it is conceivable that these debates are truly subsistent in LSD1.

Taken together, the elaborate elucidation of catalytic mechanism of LSD1 is not only of great fundamental interest, but also of high medical importance since it would facilitate the development of novel mechanism-based inactivators. Thus, in the present study, the catalytic mechanism of LSD1 was investigated by combining molecular modeling, MD simulations and QM/MM calculations. The ternary complex structure of LSD1-CoREST-substrate in aqueous solution was obtained from MD simulation. The simulation results highlight that the conserved lysine–water–flavin motif and Tyr761 may play a vital role in both properly orienting FAD with respect to substrate and expediting the demethylation process, which are consistent with the site-directed mutagenesis results and related kinetic studies. In the end, the QM/MM strategy was employed on the ternary complex structure, and the results suggest that the reductive half-reaction of LSD1 undergoes the hydride transfer process. These findings provide the atomic description of the demethylation pathway and insights into the molecular mechanism of LSD1.

## Materials and Methods

### Preparation of the simulation system

The initial configuration of the enzyme-substrate complex was modeled on the basis of the crystal structure of LSD1 in complex with CoREST, a co-repressor that enables LSD1 to demethylate nucleosomal substrates (PDB code: 2IW5) [28], and LSD1-CoREST-H3 peptide ternary complex (PDB code: 2V1D) [29]. First, the H3 peptide in ternary complex was extracted to the LSD1-CoREST complex structure which has higher resolution (R = 2.57 Å). Then, the H3 peptide was mutated to the valid substrate structure of LSD1 by replacing the mutated methionine residue with dimethylated lysine. The resulting LSD1-CoREST-substrate complex was minimized by using the AMBER force field with the following parameters: a distance-dependent dielectric function, nonbonded cutoff of 8 Å, Amber charges for the protein, and Gastieger-Hückel charges for dimethylated lysine and FAD. The structure was minimized by the simplex method, followed by the Powell method to an energy gradient <0.05 kcal/(mol·Å). All procedures were performed using the Sybyl software package (Tripos, St. Louis, MO).

### Molecular dynamics simulation

MD simulations were performed on the LSD1-CoREST-substrate complex structure. Before simulations, the protonation states of ionizable residues were chosen based on the prediction of H++ program [30] and carefully visual inspection of their local electrostatic and hydrogen bond microenvironment. The complex was solvated into a rectangular box with a 10 Å buffer distance between the solvent box wall and the nearest solute atoms. Then, the complex-water system was subjected to energy minimization. Afterward, counterions were added to the system to neutralize the simulation system and the whole system was subsequently minimized again. The charges of atoms of FAD and dimethylated lysine were calculated by using the RESP method [31] encoded in the AMBER suite of programs [32] at the level of RHF/6-31G*. Covalent and nonbonded parameters for the atoms of FAD and dimethylated lysine were assigned, by analogy or through interpolation, from those already present in the AMBER force field.

All MD simulations were performed using the AMBER package (version 10.0) with constant temperature and pressure (NPT) and periodic boundary conditions. The Amber99SB [33], [34], [35] force field for the protein and TIP3P model [36]for water molecules were employed. During MD simulations, all bonds involving hydrogen atoms were constrained with the SHAKE algorithm [37], and the integration step of 2 fs was used. Electrostatic interactions were calculated using the particle-mesh Ewald method [38]. The nonbonded cutoff was set to 10.0 Å, and the nonbonded pairs were updated every 25 steps. The simulation was coupled to a 300 K thermal bath at 1.0 atm of pressure (atm = 101.3 kPa) by applying the algorithm of Berendsen et al. [39] The temperature and pressure coupling parameters were set as 1 ps.

### QM/MM calculation

QM/MM calculations were performed with the use of a two-layered ONIOM scheme encoded in the Gaussian03 program [40]. The ONIOM method is a hybrid quantum chemical approach developed by Morokuma and coworkers that allows different levels of theory to be applied to different parts of a molecular system [41], [42], [43], [44], [45], [46], [47]. In this approach, the molecular system under investigation is defined as two parts. The “model” system consists of the most critical elements of the system and is treated with an accurate (high-level) computational method which can describe bond breaking and formation. The “real” system includes the entire system and is treated with an inexpensive (low-level) computational method which can depict the environmental effects of the molecular environment on the “model” system. The total ONIOM energy, E_ONIOM_, is defined as the following,
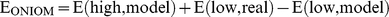
Where E(high, model) is the energy of the model system (includes the link atoms) at the high level of theory, E(low, real) is the energy of the real system at the low level of theory, and E(low, model) is the energy of the model system at the low level of theory. Thus, the ONIOM method allows one to perform a high-level calculation on just a small, critical part of the molecular system and incorporate the effects of the surrounding elements at a lower level of theory to yield a consistent energy expression on the full system.

The quantum mechanical (QM) region consists of the dimethylamino portion (-(CH_2_)_3_-N(CH_3_)_2_) of dimethylated H3K4 and the crucial parts of the conserved lysine-water-flavin motif, which includes the flavin ring and its adjacent methylene group of FAD, most of the side chain (-(CH_2_)_3_-NH2) of Lys661 and the water molecular bridging the flavin and Lys661 through two hydrogen bonds. Link hydrogen atoms [48], [49] were employed to saturate the dangling covalent bonds. The QM region comprises 66 atoms and was described in terms of the density functional theory with the B3LYP exchange-correlation functional [50], [51], [52], [53] (UB3LYP for open shell in the radical mechanism) and 6-31G* basis set. The remainder of the system (MM region) was treated by using the AMBER Parm99 force field. A total of 12984 atoms were included for the QM/MM calculations. The electrostatic interactions between the QM and MM regions were calculated by an electronic embedding scheme. The partial charges of the MM region were incorporated into QM Hamiltonian, which provides a better description of the electrostatic interaction between the QM and MM regions and allows the QM wavefunction to be polarized. The charges for all the QM atoms were fitted to the electrostatic potential at points selected according to the Merz-Singh-Kollman scheme and calculated at the B3LYP/6-31G* level. The minimized structure optimized using the AMBER Parm99 force field [34] was further optimized at the ONIOM (B3LYP/6-31G*:Amber) level.

## Results and Discussion

### The protonation states of key residues in active site

The acid dissociation constant (pKa) of ionizable residues was determined computationally by H++ program [30] on the basis of substrate-alone (histone substrate), substrate-free (LSD1-CoREST complex, PDB code: 2IW5) [28] and substrate-bound (modeled LSD1-CoREST-substrate complex, see Material and Methods) states by using different internal dielectric constants. As shown in Table 1, in the substrate-free state, the conserved Lys661 was positive-charged with a normal pKa value, whereas binding of the histone substrate within active site significantly reduced its pKa values regardless of different internal dielectric constants, which preserved Lys661 in neutral state under physiological environment. This difference can be attributed to the variation of the microenvironment in active-site. The Lys661 is located at a remarkably hydrophobic active-site channel underneath the protein surface. The exact match of the substrate with the narrow channel not only expelled solvent but also effectively sealed the active center, which was a conserved feature among flavoprotein amine oxidases [54], [55], [56]. Thus, such solvent inaccessibility of the active center gave rise to strong electrostatic effect that alters the acid/base equilibrium of Lys661. Similar results were obtained in recent studies of the catalytic mechanism of maize polyamine oxidase (MPAO) [57] and mammalian polyamine oxidase [58], two homologues of LSD1, which demonstrated the corresponding conserved lysines in active center were deprotoned upon substrate binding from both experimental and theoretical perspectives [57], [58], [59]. Furthermore, resembling Lys661, the pKa value for H3K4 in complex was remarkably lower than that of in bulk solvent. In consideration of the same niche that H3K4 and Lys661 coexisted, the transformation of acid-base property of H3K4 was likewise ascribed to the variation of the microenvironment. These results suggest that the neutral state of dimethylated H3K4 was the dominating form under physiological environment because the two methyl groups would slightly enhance the hydrophobicity of the microenvironment and then trigger more effective electrostatic effect acted on the protonation states. The neutral state of dimethylated H3K4 agrees with the observation of the neutral nitrogen at the site of oxidation in other members of monoamine oxidase family [25], [59], [60], [61], [62] and was validated by the experimental result that LSD1 preferentially bound the substrate with uncharged dimethylated H3K4 for catalysis [63]. Taken together, the conservatism of the catalytic microenvironment and the consistency between theoretical prediction and experimental results corroborate the deprotonation state of Lys661 and dimethylated H3K4 under physiological pH conditions. Accordingly, Lys661 and dimethylated H3K4 were kept uncharged during the following MD simulation and QM/MM calculation.

**Table 1:** The predicted pKa for K661 and H3K4 in three distinct states with different internal dielectric constants.

ε_in_ a	Substrate^b^	LSD1-CoREST	LSD1-CoREST-substrate	
	H3K4	K661	K661	H3K4
6	10.39	>14	0.18	<0
4	10.41	>14	<0	<0
2	10.42	>14	<0	0.77

a Internal dielectric constant.

b H3 peptide in bulk solvent.

### The 3D model of LSD1-CoREST-substrate complex

**Figure 3:**
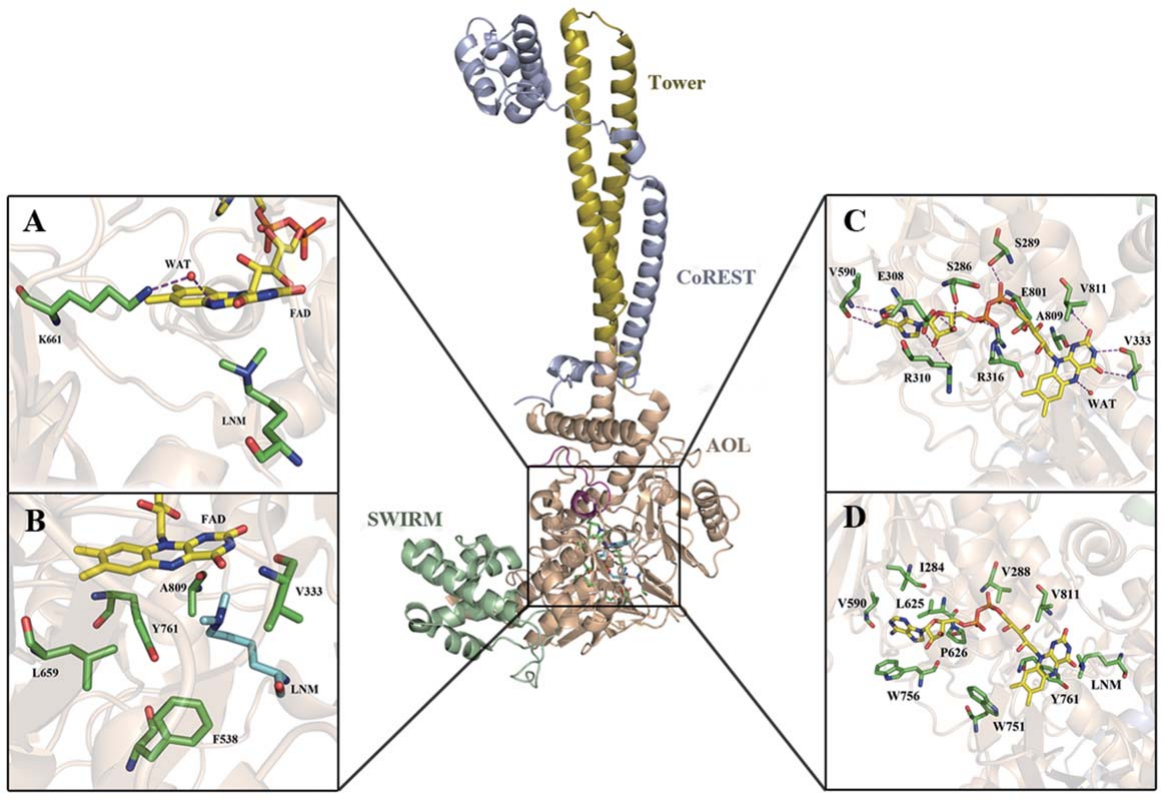
The overall structure of LSD1-CoREST-substrate complex and key interactions in the catalytic chamber. Cartoon diagram of the LSD1-CoREST-substrate complex highlights the SWIRM domain (green), AOL domain (wheat), Tower domain (golden yellow), CoREST (light blue) and substrate peptide (magenta). (A) H-bond interactions in the conserved lysine-water-flavin motif. The bridging water molecular is shown in red sphere and the residues and FAD are shown in green and yellow sticks, respectively. H-bonds are indicated with purple dashed lines. (B) Hydrophobic interactions of dimethylated H3K4 with its surrounding residues in the catalytic chamber. Dimethylated H3K4 is shown in cyan for the sake of clarity. (C) H-bond interactions of FAD with its surrounding residues. (D) Hydrophobic interactions of FAD with its surrounding residues.

Structurally, LSD1 polypeptide chain folds into a highly asymmetric configuration with three distinct functional domains, as shown in Figure 3 (middle panel). The N-terminal SWIRM (Swi3p, Rsc8p and Moira) domain, which is found in several chromatin-associated proteins, adopts a completely helical histone fold. It closely packs against the C-terminal AOL (amine oxidase-like) domain in which the demetylation reaction takes place. The AOL domain exhibits a significant homologous topology to the monoamine oxidase family and contains a typical large insertion that adopts a tower-like structure (Tower domain) with two antiparallel helices interacting with CoREST.

**Figure 4:**
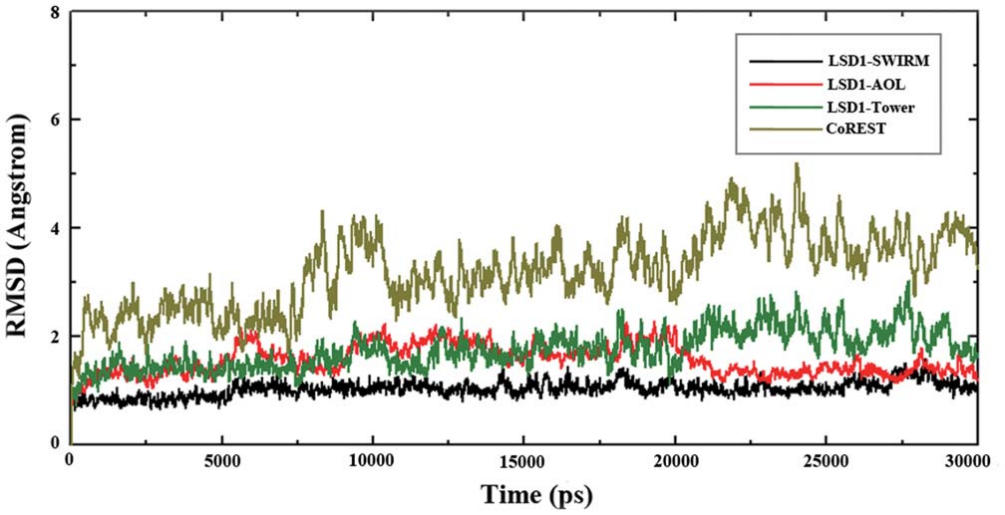
Time dependencies of the weighted root-mean-square deviations for the backbone atoms of the three domains of LSD1 and CoREST from their initial positions during the 30-ns simulation.

To investigate the stability of the active site cavity, a 30-ns MD simulation was performed on the LSD1-CoREST-substrate complex. The temporal development of the weighted root-mean-square deviations (RMSD) for the atoms of different domains of LSD1 and CoREST from their initial positions (t = 0) were monitored. As illustrated in Figure 4, the steady RMSD for the atoms in the three major functional domains of LSD1, especially for the catalytic AOL domain, indicated that the catalytic cavity is relatively stable during the MD simulation and the trajectory of the MD simulation for the LSD1-CoREST-substrate ternary complex is reliable for post analysis.

**Figure 5:**
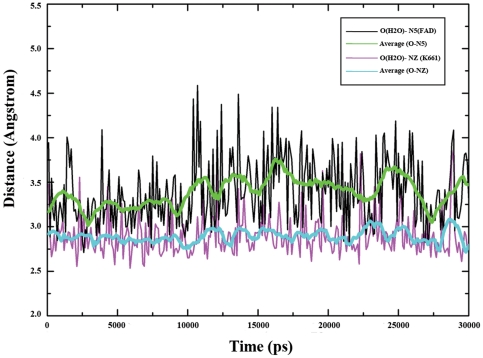
Distance evolution along simulation time for the two hydrogen bonds in the lysine-water-flavin motif. The red curve and blue curve were obtained by 5 ps averaged.

**Figure 6:**
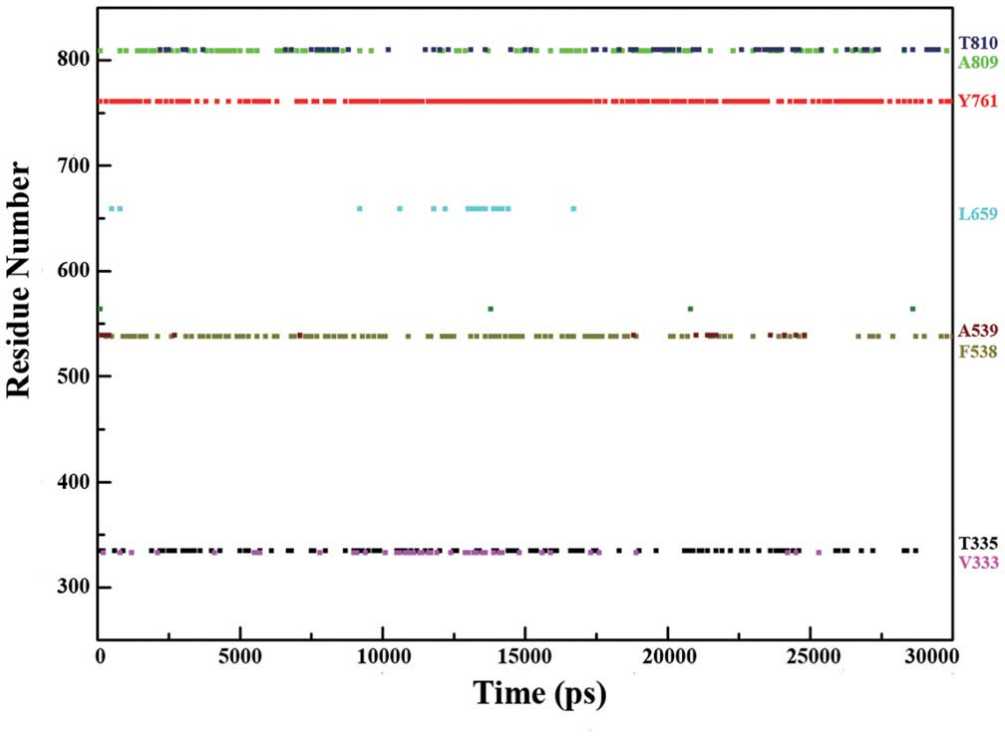
The residues involved in hydrophobic interactions with dimethylated H3K4 versus simulation time. Different colors are used for the sake of clarity.

To probe the molecular basis for demeylation catalysis, both the H-bond and hydrophobic interactions in the active center were analyzed. In all, the H-bonds amongst LSD1, FAD and histone substrate were mostly maintained during the 30-ns MD simulation as indicated by their occupancies (Table S1), which further indicated the stability of the demethylation environment during MD simulation. By the distance evolution monitoring of the region neighboring the flavin ring of FAD, a key lysine was identified to interact with the N5 atom of FAD via a water molecule by two H-bonds (Figure 3A), which characterized the conserved lysine-water-flavin motif. In addition, as shown in Figure 5, the two H-bonds were mostly conserved during MD simulation, with an average interaction distance about 3.3 and 3.0 Å for O(H2O)-N5(FAD) and O(H2O)-NZ(K661), respectively. Therefore, it can be inferred that the stable H-bond interactions in lysine-water-flavin motif effectively maintained FAD in a reactive conformation and further facilitated the biological reaction through polarizing the N5 atom in FAD. This hypothesis was in agreement with the mutagenesis study that the disruption of this conserved H-bonds interaction pattern with the K661A mutation in LSD1 completely abolished its demethylation activity [64] and the substitution of the corresponding Lys300 with Met in MPAO resulted in a 1400-fold decrease in the rate of flavin reduction [57]. Meanwhile, Baron et al [65] recently reported that, during the oxidative half-reaction, K661 may function as a “entry residue” to channel the oxygen molecule to the catalytic chamber and contribute to oxygen activation through stabilizing the superoxide-flavin semiquinone intermediates, which further highlighted the crucial role of K661 in demethylation and was compatible with our simulation results. Furthermore, the hydrophobic interactions between dimethylated H3K4 and the hydrophobic catalytic chamber (Figure 3B) of LSD1 were plotted along the simulation time. As shown in Figure 6, nearly all of the residues in the catalytic chamber were involved in the hydrophobic interactions with dimethylated H3K4, viz., Y761, F538, T810, A809, V333, T335, A539 and L659, strongly supporting the assumption that the hydrophobic interaction was a non-trivial driving force for substrate binding and emphasizing the importance of each residue in the catalytic chamber for the precise positioning of the methylated lysine with respect to the flavin ring. This result was fully consistent with the experimental results that any mutation in catalytic chamber would severely impair the enzyme activity [64]. Remarkably, the aromatic side chain of Tyr761, which was highly conserved for consisting of the so-called “aromatic cage” in the monoamine amine oxidase family [66], [67], formed the most stable interactions with the dimethylamino group of H3K4, implying a more fundamental role of Tyr761 in positioning the substrate in a productive binding orientation for catalytic oxidation. In sum, both the lysine-water-flavin motif and Tyr761 are deemed to be the crucial factors for maintaining the optimum configurations between FAD and dimethylated H3K4. The synergy of the two factors stabilized the catalytic environment and facilitated the biochemical reaction via both structural and electrostatic effect. Taken together, the consistence between simulation results and the experimental data demonstrated that the MD simulation on LSD1-CoREST-substrate complex is reasonable and our ternary complex model is reliable for further study.

### Sampling from MD simulations

A proper sampling methodology is pivotal to the reliability of QM/MM calculations. Fraaije MW et al [56] thoroughly explored the recurrent features in catalytic apparatus of different flavoproteins by carefully inspecting their three-dimensional structures. They pointed out the essential stereochemistry principles underlying the dehydrogenation reactions: (1) the H-bond donor within the catalytic chamber is located on the flavin side opposite to that facing the substrate; (2) the angle between N10, N5 and the hydrogen-bond donor ranges from 116°to 170°; (3) the site of oxidative attack typically binds in front of the flavin at 3.5 Å distance from N5; (4)the angle defined by the site of oxidative attack with the N5–N10 atoms has a narrow range of 96–117°. Furthermore, taking into account the extent of fluctuation of RMSD value during MD simulation, the snapshots in the equilibrium state since 10 ns were treated with these statistical criterions. Configurations fulfilling these criterions were extracted to to select a proper starting point for subsequent QM/MM study. As shown in Figure S1 in Supporting Information, the binding mode and conformation of FAD in the selected snapshot closely resemble those observed in other flavinenzymes [54], [68], [69]. The interaction analysis (Figure 3C, 3D) based on the snapshot indicates the hydrophobic interactions with FAD were highly conserved and quite compatible with the previous experimental study [70], conforming this snapshot was a reasonable initial structure for the QM/MM study.

### QM/MM Results

#### Direct hydride transfer mechanism

To explore the underlying feasibility of the reductive half-reaction along the three debated proposals, the direct hydride transfer mechanism was firstly investigated with the QM/MM strategy. The system for QM/MM simulation was constructed based on the aforementioned snapshot. The QM region was composed of the dimethylamino portion (-(CH_2_)_3_-N(CH_3_)_2_) of dimethylated H3K4 and the isoalloxazine ring with its adjacent methylene group of FAD. Furthermore, considering the vital roles of lysine-water-flavin motif in demethylation, the majority of the side chain (-(CH_2_)_3_-NH_2_) of Lys661 and the water bridging it with flavin were also included in the QM region. The remainder of the complex was included in the MM region. The partitioning scheme for QM and MM regions is described in the Materials and Methods section. We designate this structure as a reactant system. ONIOM, a QM/MM method encoded in Gaussion03, was utilized for all the QM/MM calculations.

**Figure 7:**
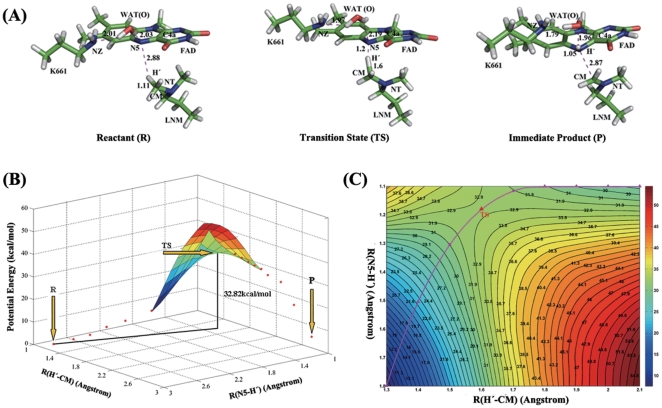
The potential energy surface of the reductive half-reaction in the demethylation of LSD1-CoREST-substrate complex. (A) The QM/MM optimized structures of the reactant (R), transition state (TS), and immediate product (P). For clarity, only the structures in the QM region have been shown. (B) The potential energy surface (PES) of the reductive half-reaction along the defined reaction coordinates. (C) Contour plot of the PES corresponding to the central part in (B). The pink triangle line represents the lowest energy pathway on the calculated PES and the position of the TS is marked by a red triangle.

The QM/MM optimized geometry of the reagent system varied slightly with the initial structure obtained by MD simulation. The distance from the oxygen atom of conserved water molecule to the N5 atom of FAD was 2.93 Å with that the angle consisted of O-H and N5 was 175.49°, which indicated a strong H-bond was involved in the catalytic center. The angle between N10, N5 and the hydrogen-bond donor was 136.90°. Meanwhile, the site of oxidative attack bound in front of the flavin at a distance of 3.70 Å from N5, and defined the angle with the N5–N10 atoms at 111.42°. The optimized configuration was quite compatible with the stereochemistry principles for productive binding of flavoenzymes [56], demonstrating that the model was reliable for the QM/MM calculation. Along the reaction scheme of the direct hydride transfer mechanism, the energies of the reagent (R), transition state (TS), and immediate product (P) were determined by two-dimensional QM/MM potential energy surface by defining the distances of R(H′-CM) and R(N5-H′) as the reaction coordinates (Figure 7B,C). In the optimized reagent, R(H′-CM) = 1.11 Å and R(N5-H′) = 2.88 Å; while in the optimized immediate product, the H′-CM bond is broken (R(H′-CM) = 2.87 Å) and the N5-H′ bond is formed(R(N5-H′) = 1.05 Å). The calculated potential energy barrier of Proposal 1 is ΔE^≠^ = 32.82 kcal/mol, which is slightly higher than that of other flavoenzymes, such as MTOX that the calculated potential energy barrier for hydride transfer mechanism is about 27.4 kcal/mol. [71] This higher potential energy barrier is quite in agreement with the experimental results that the rate constant for the oxidation of substrate of LSD1 is 2–5 orders of magnitude slower than values reported for other flavoprotein oxidases [63].

The structure of the transition state (TS) in Proposal 1 was determined by adiabatic mapping at the QM/MM level (Figure 7A). In the TS, R(H′-CM) = 1.6 Å and R(N5-H′) = 1.2 Å, and the dimethylamino group tends to be in a plane concomitant with the contraction of the CM-NT bond. This structural reorganization clearly illustrated that the N5-H′ bond was partially formed while the H′-CM bond was partially broken, and indicated that the reductive half-reaction belonged to the direct hydride transfer mechanism. The overall reaction is calculated to be endothermic by ΔE = 5.44 kcal/mol. Furthermore, as the decrease of the distance between N5 and H′, the H′-CM bond elongated to cleavage spontaneously (Figure S2), the bond order of CM-NT was transformed from single to double in the intermediate product, which was in agreement with the direct hydride transfer mechanism. On the basis of the one-dimensional potential energy profile (Figure S2), the transition state was located as R(H′-CM) = 1.43 Å and R(N5-H′) = 1.2 Å with the energy barrier about 32.71 kcal/mol. Accordingly, the results further proved the reductive half-reaction in the demethylation process belonged to the direct hydride transfer mechanism, which coincided with recent findings from the exquisite kinetic isotope effects experiments on different flavoprotein oxidases [25], [71], [72], [73], [74], [75], [76], [77], [78].

**Figure 8:**
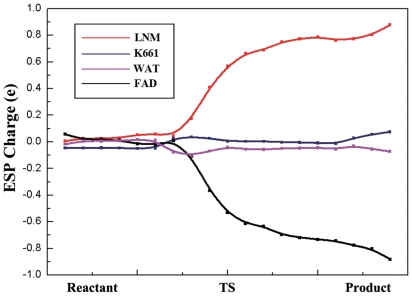
ESP charge distributions of the four groups in the QM region along the hydride transfer reaction.

In the redox catalysis, the consumption of the substrates was often accompanied with the charge transfer and the coinstantaneous change of redox states. And during the reductive half-reaction of LSD1, FAD was reduced to FADH^−^ by a hydride equivalent that transferred from the dimethylated H3K4 and resulted in a positive charged imine intermediate. To further characterize the charge transfer in the reductive half-reaction of LSD1, the evolution of the electrostatic potential (ESP) derived charges of all pieces in the QM region were monitored (Figure 8). As expected, the overall ESP charges of Lys661 and water molecular remained approximately constant throughout the reaction, consistent with the fact that they were not directly involved in the hydride transfer pathway (Figure 2). Whereas the apparent proportional variations between the ESP charges of FAD and substrate were clearly observed. In the reactant system, both FAD and substrate were uncharged. Then the positive charge on substrate progressively increased until the formation of the imine cation intermediate. Meanwhile, the negative charge increasingly aggregated on FAD which resulted in the reduced cofactor with a negative net charge. The results definitely indicated that accompanying with the migration of the hydrogen atom from CM to N5, a pair of electrons concomitantly transferred from the substrate to FAD, which was fully compatible with the direct hydride transfer mechanism and in good agreement with previous theoretical studies of the catalytic mechanism of D-Amino acid oxidase (DAAO) [79].

#### Other mechanisms

To investigate the conformational change of the catalytic region along the polar nucleophilic mechanism, the NT-C4a bond was constrained and curtailed from 3.5 to 1.5 Å, and the full optimization was performed for the remainder. Unfortunately, the intermediate substrate-FAD adduct, served as a hinge for the polar nucleophilic mechanism, was not obtained in our studies. Furthermore, the feasibility of the radical mechanism for demethylation reaction was also investigated. The notable high potential energy barrier (ΔE^≠^>40 kcal/mol) for the generation of the aminium radical was predicted to severely impede the following reactions, in good accordance with the theoretical results of MTOX that an unreasonable high energy barrier was necessitated for the radical mechanism [71]. Meanwhile, as α-CH bond cleavage has been experimentally proven to be the rate-limiting step, there is unlikely such an unfavorable energy barrier preceded this step. Therefore, our QM/MM results support the hypothesis that the requisite intermediates prior to CH bond cleavage for the two mechanisms could rarely exist due to their unfavorable thermodynamic properties [80], and agree well with the experimental evidence that no intermediates between oxidized and reduced flavin were detected [63], providing indirect supports to the direct hydride transfer mechanism.

### Conclusions

LSD1 is the first identified lysine-specific histone demethylase that can specifically demethylate mono- or dimethylated histone H3 lysine4 (H3-K4) and H3 lysine 9 (H3-K9) via a redox process. It plays a vital role in cell proliferation, adipogenesis, spermatogenesis, chromosome segregation and embryonic development. As a potential drug target for discovering anti-tumor drugs, the medical importance of this enzyme has also been greatly appreciated. However, the catalytic mechanism of LSD1 demethylation reaction remains ambiguous, in particular a heated controversy still lie in the rate-limiting reductive half-reaction. Therefore, in the present study, we focused on the reductive half-reaction of the demetylation reaction in LSD1-CoREST-substrate complex system, and theoretically confirmed the catalytic mechanism of this step belongs to the direct hydride transfer mechanism.

Firstly, by using molecular modeling and theoretical titration methods, the 3D structural model of LSD1-CoREST-substrate complex was constructed. Then MD simulations were performed on the structure model and a representative structure was sampled from the MD trajectory. The validity of the structural model was confirmed based on the consistency between simulation results and available experimental mutagenesis and enzymatic data for LSD1. The H-bonds and hydrophobic interaction analysis highlighted the conserved lysine-water-flavin motif and Tyr761 in the catalytic chamber. The synergy of the two factors stabilized the catalytic environment and contributed to the optimal orientation between FAD and dimethylated H3K4. Meanwhile, the pivotal hydrogen bond interaction pattern in lysine-water-flavin motif was deemed to facilitate the demethylation reaction by elaborate electrostatic interactions.

Secondly, the QM/MM calculations were performed on the representative complex structure. In addition to the groups directly participated in the reaction, Lys661 and its bridging water molecular in the lysine-water-flavin motif were also incorporated into the QM region in consideration of their non-trivial role in demetylation reaction. The 2D QM/MM potential energy surface with the located transition state of R(H′-CM) = 1.6 Å and R(N5-H′) = 1.2 Å indicated that the reductive half-reaction intrinsically belongs to the direct hydride transfer mechanism. In addition, this data was further validated by our charge distribution analysis along the reaction coordinate. In concert with the absence of detectable intermediates between oxidized and reduced FAD and the unfavorable energetics for the other two possible mechanism proposals, our studies suggested that the rate-limiting reductive half-reaction of LSD1 employed the direct hydride transfer mechanism. Accordingly, our research provided a detailed mechanism elucidation for the reductive half-reaction of demethylation by LSD1, explored the molecular basis of the demethylation pathway, and shed light on the discovery of novel mechanism-based modulators for LSD1.

## Supporting Information

Table S1 **Hydrogen bonds existing in the LSD1-CoREST-Substrate complex and their occupancies in the 30-ns MD simulation.**
(DOC)

Figure S1 **Local conformation of residues around FAD binding site in the superimposed structures obtained from the sampled snapshot of LSD1 and the crystal structures of homologous flavinenzymes (1GOS (human MAO B), 2VVM (Aspergillus niger MAO N) and 1B5Q (Zea mays PAO)).** The carbons in LSD1, 1GOS, 2VVM and 1B5Q are colored by green, cyan, yellow and pink, respectively.
(DOC)

Figure S2 **The one-dimensional potential energy profile and the corresponding R(H′-CM) distance profile along the reaction path obtained by defining the distance of R(N5-H′) as the reaction coordinate.**
(DOC)
