# Supplementary material for: Catalytic Mechanism Investigation of Lysine-Specific Demethylase 1 (LSD1): A Computational Study
Source: PLoS One. 2011 Sep 30;6(9):e25444. doi: 10.1371/journal.pone.0025444 (PMC3184146; doi:10.1371/journal.pone.0025444)
Supplement: Table S1 — Hydrogen bonds existing in the LSD1-CoREST-Substrate complex and their occupancies in the 30-ns MD simulation. (DOC) [file pone.0025444.s001.doc]

**Supporting Information**

**Table S1.** Hydrogen bonds existing in the LSD1-CoREST-Substrate complex and their occupancies in the 30-ns MD simulation.

| No. | Hydrogen Bond | Hydrogen Donor | Hydrogen Acceptor | Occupancy in 30ns-MD simulation |
| --- | --- | --- | --- | --- |
| 1 | LSD1-FAD | FAD: O3B | Glu308:OE1 | 100 |
|  | | FAD: O3B | Glu308:OE2 | 100 |
|  |  | FAD: O2B | Glu308:OE2 | 100 |
|  |  | FAD:N3 | Val333:O | 100 |
|  |  | FAD:N6A | Val590:O | 100 |
|  |  | Arg310:NE | FAD:O3B | 99.95 |
|  |  | Arg310:NE | FAD:O2B | 99.97 |
|  |  | Arg310:NH2 | FAD:O2B | 99.94 |
|  |  | Ala309:N | FAD:N3A | 99.98 |
|  |  | Val590:N | FAD:N1A | 98.99 |
|  |  | Arg316:NE | FAD:O1A | 98.15 |
|  |  | Arg316:NH2 | FAD:O1A | 97.53 |
|  |  | Arg316:N | FAD:O2A | 91.61 |
|  |  | Ser289:N | FAD:O1P | 90.23 |
|  |  | Ser289:OG | FAD:O1P | 90.07 |
|  |  | Glu801:N | FAD:O2P | 76.58 |
|  |  | Val811:N | FAD:O2 | 74.25 |
|  |  | Val333:N | FAD:O4 | 72.72 |
| 2 | LSD1-Substrate | H3Ala1:N | Asp555:OD2 | 94.71 |
|  | | ASN540:ND2 | H3Ala1:O | 81.65 |
|  |  | H3Arg2:NE | Asp553:OD1 | 90.80 |
|  |  | H3Arg2:N | Asp556:OD1 | 100 |
|  |  | H3Gln5:N | H3Arg2:O | 95.22 |
|  |  | H3Thr3:N | Asp556:OD1 | 100 |
|  |  | H3Thr3:OG1 | Asp556@OD1 | 99.21 |
|  |  | His564:NE2 | H3Thr3:O | 96.89 |
|  |  | Asn535:ND2 | H3Ala7:O | 98.81 |
|  |  | H3Arg8:NE | Asp360:O | 91.22 |
|  |  | H3Arg8:NH1 | Asp375:OD1 | 88.76 |
|  |  | H3Lys9:NZ | Glu559:OE2 | 76.71 |
|  |  | H3Arg2:NE | H3Ser10:OG | 70.48 |
|  |  | H3Ser10:OG | H3Gln5:OE1 | 83.21 |
|  |  | H3Thr11:N | Asn383:OD1 | 93.69 |
|  |  | H3Thr11:OG1 | Asn383:ND2 | 88.81 |
|  |  | H3Gly12:N | Glu387:OE1 | 96.24 |
|  |  | H3Gly13:N | Asn383:OD1 | 91.87 |
|  |  | H3Lys14:N | Glu387:OE2 | 77.6 |
|  |  | H3Lys14:NZ | Glu559:OE1 | 87.58 |
