# Supplementary material for: Catalytic Mechanism Investigation of Lysine-Specific Demethylase 1 (LSD1): A Computational Study
Source: PLoS One. 2011 Sep 30;6(9):e25444. doi: 10.1371/journal.pone.0025444 (PMC3184146; doi:10.1371/journal.pone.0025444)

**Figure S1.** Local conformation of residues around FAD binding site in the superimposed structures obtained from the sampled snapshot of LSD1 and the crystal structures of homologous flavinenzymes (1GOS (human MAO B), 2VVM (Aspergillus niger MAO N) and 1B5Q (Zea mays PAO)). The carbons in LSD1, 1GOS, 2VVM and 1B5Q are colored by green, cyan, yellow and pink, respectively.


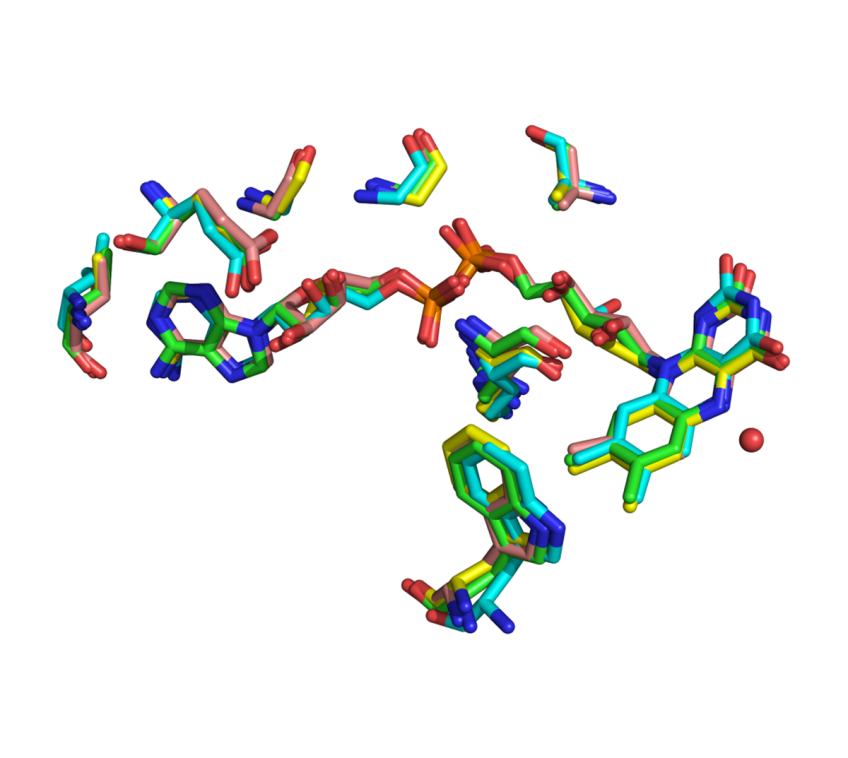

Supplement: Figure S1 — Local conformation of residues around FAD binding site in the superimposed structures obtained from the sampled snapshot of LSD1 and the crystal structures of homologous flavinenzymes (1GOS (human MAO B), 2VVM (Aspergillus niger MAO N) and 1B5Q (Zea mays PAO)). The carbons in LSD1, 1GOS, 2VVM and 1B5Q are colored by green, cyan, yellow and pink, respectively. (DOC) [file pone.0025444.s002.doc]
