# Supplementary material for: Catalytic Mechanism Investigation of Lysine-Specific Demethylase 1 (LSD1): A Computational Study
Source: PLoS One. 2011 Sep 30;6(9):e25444. doi: 10.1371/journal.pone.0025444 (PMC3184146; doi:10.1371/journal.pone.0025444)

**Figure S2.** The one-dimensional potential energy profile and the corresponding R(H´-CM) distance profile along the reaction path obtained by defining the distance of R(N5-H´) as the reaction coordinate.


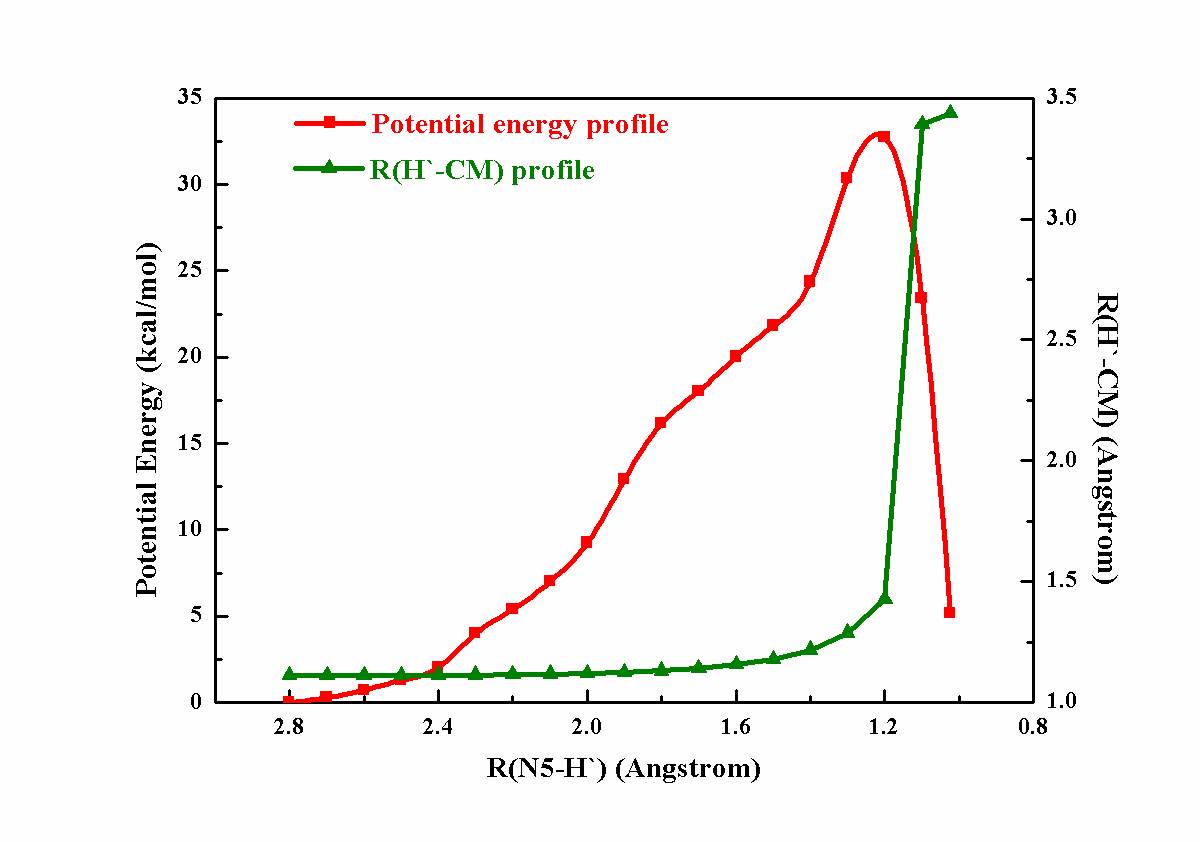

Supplement: Figure S2 — The one-dimensional potential energy profile and the corresponding R(H′-CM) distance profile along the reaction path obtained by defining the distance of R(N5-H′) as the reaction coordinate. (DOC) [file pone.0025444.s003.doc]
